# Structural analysis and ionic conduction mechanism of sulfide-based solid electrolytes doped with Br

**DOI:** 10.1038/s41598-023-43347-9

**Published:** 2023-09-25

**Authors:** Hiroshi Yamaguchi, Kentaro Kobayashi, Satoshi Hiroi, Futoshi Utsuno, Koji Ohara

**Affiliations:** 1 Graduate School of Natural Science and Technology, 1060, Nishikawatsu-Cho, Matsue, Shimane 690-8504 Japan; 2 Idemitsu Kosan Co. Ltd., 1280, Kamiizumi, Sodegaura-City, Chiba 299-0293 Japan; 3 Faculty of Materials for Energy, Shimane University, 1060, Nishikawatsu-Cho, Matsue, Shimane 690-8504 Japan; 4 Diffraction and Scattering Division, Japan Synchrotron Radiation Research Institute, 1-1-1, Kouto, Sayo-Cho, Sayo-Gun, Hyogo 679-5198 Japan

**Keywords:** Batteries, Glasses

## Abstract

Sulfide glasses can exhibit notable ionic conductivity because of annealing-associated crystallization. One well-known example is Li_7_P_3_S_11_. Our research showed that adding bromine (Br) to Li_3_PS_4_ sulfide glass results in a similar crystal structure and high ionic conductivity comparable to that of another compound Li_10_GeP_2_S_12_. This structure differs from the PS_4_ anion framework of Li_3_PS_4_. In addition, the ionic conductivity decreases owing to a structural transition to the β-phase. Herein, we present our findings on the local structure of Li_3_PS_4_ sulfide glass and its crystallized glass ceramic with the addition of Br. This analysis relies on the pair distribution function analysis obtained from high-energy X-ray diffraction. Moreover, using the bond valence sum method, we verified that incorporating Br promotes the formation of Li ionic conduction pathways. Our results indicate that precise control over the anion molecular structure by introducing halogens holds promise for achieving high Li-ion conductivity.

## Introduction

One of the key strategies to achieve carbon neutrality is the development of all-solid-state batteries. Conventional liquid batteries utilize flammable organic solvents as electrolytes, raising safety concerns such as leakage and resulting ignition. In contrast, the electrolyte in an all-solid-state battery is solid, effectively addressing the safety issues associated with liquid-based batteries. Moreover, all-solid-state batteries can outperform liquid batteries in practical applications, especially in the automotive and stationary sectors. They offer advantages such as a high lithium-ion transport ratio and high energy output^1,2^, enabling high voltage operation and enhanced capacity. Additionally, all-solid-state batteries can utilize high-capacity sulfur-based electrode materials^3^, which are incompatible with liquid electrolytes owing to their high reactivity with solvents^4^. As a result, extensive research is underway to explore and advance the potential of all-solid-state batteries in achieving these desirable features^5,6^.

One of the crucial components in the advancement of all-solid-state batteries is the solid electrolyte. Among various solid electrolytes, the sulfide-based systems^7–13^ exhibited high ionic conductivity. The Li_2_S–P_2_S_5_ system demonstrates different glassy and crystalline structures depending on the composition and heat treatment temperature. In the Li_2_S:P_2_S_5_ = 75:25 system, the glass exhibits a conductivity of ~ 10^−4^ S/cm, while the beta crystals and other structures in the high-temperature range have conductivity < 10^−4^ S/cm, similar to that of glass. In contrast, the Li_2_S:P_2_S_5_ = 70:30 system exhibits Li_7_P_3_S_11_ crystal with a remarkable ionic conductivity of 3.2 × 10 mS/cm^14,15^. However, this system contains P_2_S_7_ anions, so H_2_S generation is more likely to occur^16^. To address the H_2_S generation issue, researchers have developed solid electrolytes comparable to liquid electrolytes. An example of such a solid electrolyte is Li_10_GeP_2_S_12_, commonly known as LGPS, developed by Kanno, which exhibits a conductivity of 12 mS/cm^17–19^. Li_9.54_Si_1.74_P_1.44_S_11.7_Cl_0.3_ is another important solid electrolyte with a conductivity of 25 mS/cm^20^. These systems do not contain the P_2_S_7_ anions. However, these materials rely on the costly rare metal Ge and face challenges in terms of electrical stability. As a result, alternative materials using Sn or Si instead of Ge^21,22^ and designs incorporating O in place of S have also been explored^23^.

High ionic conductivity and the absence of rare metals are crucial for the industrial implementation of sulfide-based solid electrolytes. Herein, we have successfully developed a solid electrolyte material with high ionic conductivity by incorporating halogen elements into Li_3_PS_4_ glass, which serves as the base material^24–28^. In this study, we present a comprehensive analysis of the detailed structure of the developed material.

## Experimental

### Material synthesis

Li_2_S (> 99.9%, Idemitsu Kosan, Japan), P_2_S_5_ (> 99%, Sigma-Aldrich Japan, Japan), and LiBr (> 99.99%, Fujifilm Wako Pure Chemicals Corporation, Japan) were used as raw materials. These materials were carefully weighed and mixed in a glove box under an argon atmosphere with a dew point of − 80 °C. The aim was to obtain 10 g of each material, following the composition ratios shown in Table 1. The powders and ten zirconia balls with a diameter of 10 mm were added to a fully sealed alumina pot and placed in a planetary ball mill (Fritsch: Model No. P-7).

**Table 1:** Sample composition ratios and ionic conductivities.

Sample	Li_2_S	P_2_S_5_	LiBr	Ionic conductivity (mS/cm) in this work	Ionic conductivity (mS/cm) in previous works
Pure LiPS glass	75	25	0	0.39	0.15^12^, 0.51^24^
Br-doped glass	63.8	21.3	15	0.52	0.53 ~ 0.61^24^
Br-doped glass ceramic	63.8	21.3	15	3.2	2.5^24^

Initially, the planetary ball mill was operated at a low speed of 100 rpm for a few minutes to facilitate mixing. The rotation speed was then gradually increased to 370 rpm. Mechanical milling was conducted at 370 rpm for 20 h to synthesize the glass. The resulting glass was subjected to heat treatment at various temperatures to obtain glass ceramic, as listed in Table 1. The samples were treated at the desired temperature for 2 h under an argon atmosphere and then cooled to room temperature.

### Characterization

This study examines the powder X-ray diffraction (XRD) measurements using a Smart Lab instrument (Rigaku, Japan). The diffraction angle (2θ) range of 10°–60° was scanned with a step size of 0.02° at room temperature (290–300 K). Before measurement, we meticulously positioned each sample in a glass holder with a smooth surface inside a glove box under an argon atmosphere, maintaining precise control over the dew point. The glass holder was then sealed using Kapton film tape.

High-energy X-ray scattering measurements using synchrotron radiation were carried out at BL04B2 of SPring-8^29,30^. The X-ray system employed incident energy of 61.339 keV, consisting of four CdTe and two Ge detectors. To enable X-ray diffraction, we hermetically sealed all samples in 2.0-mmΦ borosilicate glass capillaries (WJM-Glas/Muller GmbH) inside a glove box under an argon atmosphere with precise dew point control. The required scattering intensities *I*(*Q*) were meticulously corrected for background, Compton scattering, polarization, and absorption. Subsequently, the structure factor *S*(*Q*) was calculated, and the reduced pair distribution function *G*(*r*) was obtained using the Fourier transform of *S*(*Q*).

Density functional theory structure optimization calculations were performed using BLYP/GGA with DMol^3^ from Material Studio^31,32^. Structural refinement was conducted on laboratory X-ray diffraction (lab-XRD) data using the Rietveld refinement method^33^. The calculation of Li conduction paths was accomplished using the SoftBV^34–36^. VESTA was used for the three-dimensional display of the paths^37^.

## Results and discussion

**Figure 1:**
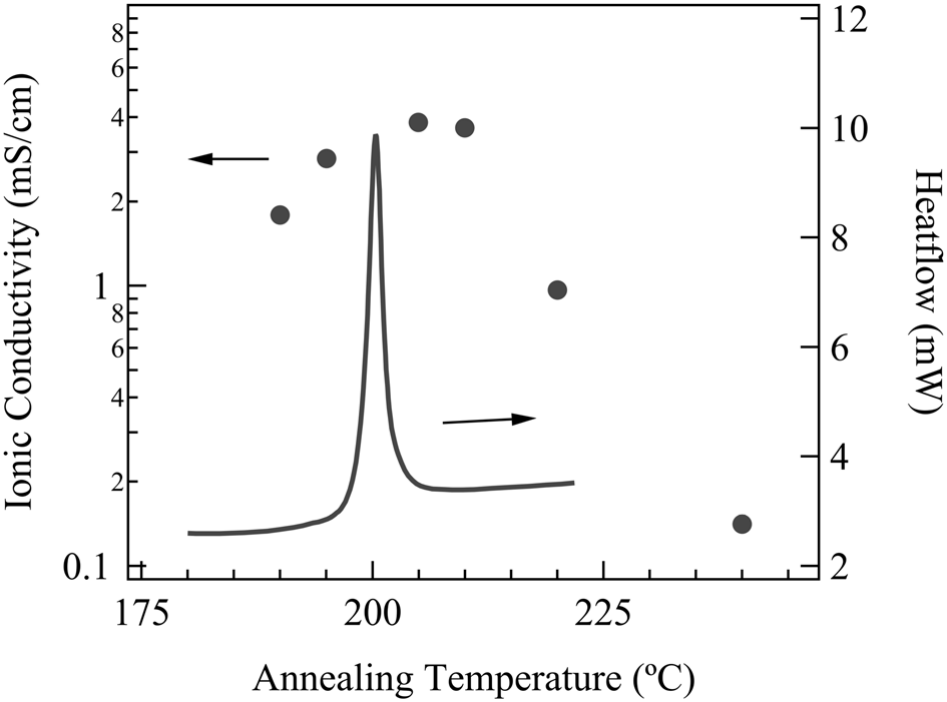
Heat flow of the glassy LiPS-Br annealed to 220 °C and the ionic conductivity at each annealing temperature.

**Figure 2:**
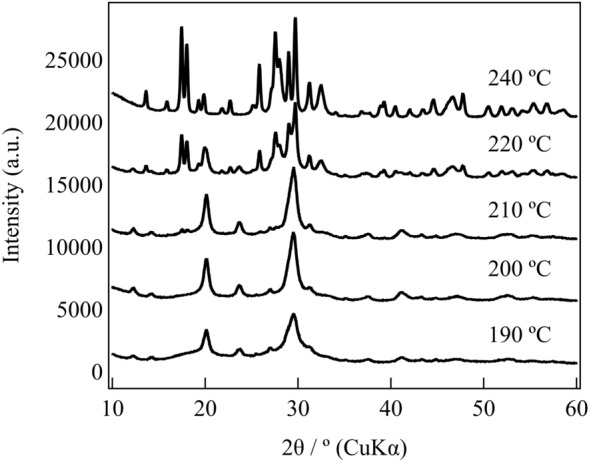
XRD patterns at each annealing temperature.

Figure 1 depicts the heat flow behavior of glassy LiPS-Br annealed at 220 °C and the corresponding ionic conductivity at each annealing temperature. The XRD patterns shown in Fig. 2 reveal that within the temperature range of 190 °C–210 °C, a previously unidentified crystalline phase emerged as the primary source of ionic conductivity, surpassing 1 mS/cm. Conversely, when the temperature exceeded 220 °C, β-crystals became evident, considerably decreasing ionic conductivity. The newly observed unknown phase is presumed to possess a metastable structure, evident from its broad half-width and limited temperature range within a single phase.

**Figure 3:**
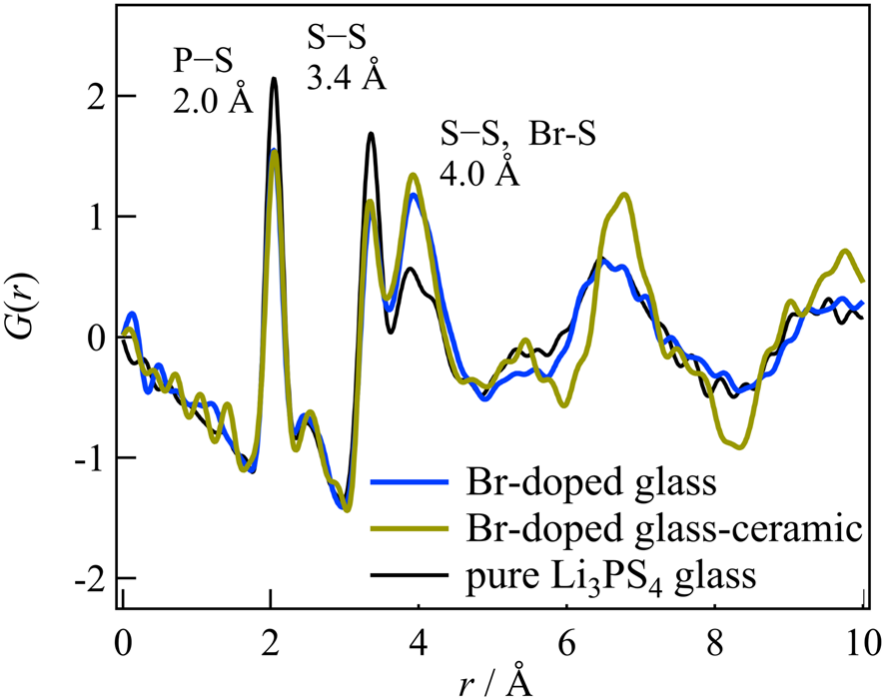
Comparison of Br-doped glass and glass ceramic with a pure Li_3_PS_4_ glass as a reference.

Figure 3 illustrates the experimental X-ray pair distribution function for the glass and the crystal after annealing at 200 °C, with the Li_3_PS_4_ glass serving as a reference. A comparison of the glass structures reveals a P–S correlation within the PS_4_ molecule at a distance of 2.0 Å, an S–S correlation within the same molecule at 3.4 Å, and a peak at ~ 4.0 Å originating from an S–S correlation between the molecules^8^. Adding Br does not significantly affect the glassy structure, as the peak positions and pair distribution functions (PDFs) above 5 Å remain almost identical before and after adding Br, despite variations in peak intensities caused by compositional differences. The area ratio of the P–S correlation located at 2.0 Å and the X-ray weight factor ratio was calculated to be 0.754 and 0.758, respectively. The difference in peak intensity can be attributed to compositional disparities, but there is no notable distinction in the P–S correlation or the presence of PS_4_ molecules within each glass structure. Adding Br increases the intensity of the peak located at 4.0 Å, indicating the presence of a correlation associated with Br. Comparing the Br added glass and crystal, no changes are observed in the P–S correlation within the PS_4_ molecule appeared at 2.0 Å or the S–S correlation within the same molecule located at 3.4 Å, confirming that no alterations have occurred within the PS_4_ molecule from glass to the crystal. However, the crystals exhibit an intensity increase in the peak observed at ~ 4.0 Å, corresponding to the S–S distance between PS_4_ molecules. Based on the observed correlation related to Br at this distance, it can be deduced that alternations occur in the correlation between PS_4_ molecules, between PS_4_ molecules and Br^−^ anions, or among the Br^−^ anions them can be inferred, given the correlation related to Br at this distance. Furthermore, a peak indicative of the ordering of the molecular arrangement is observed in the crystal above 5.0 Å.

**Figure 4:**
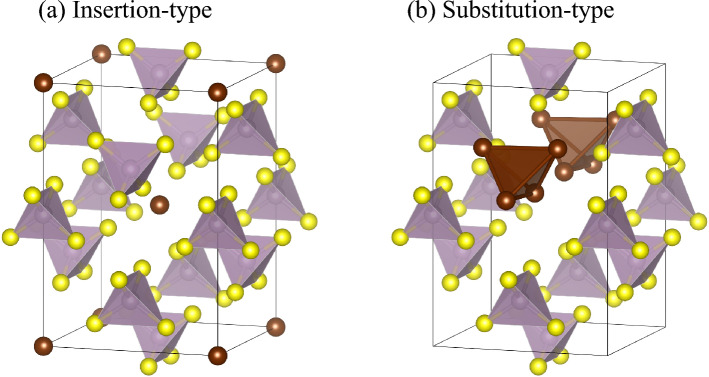
Insertion type (**a**) and substitution-type (**b**) structural models of Br-doped glass ceramic.

Herein, we investigate the high ionic conductivity observed in Br-doped glass ceramic, which exhibit comparable performance to Li_7_P_3_S_11_, comprising PS_4_ and P_2_S_7_ molecules. Therefore, we determined the specific location of the Br^−^ anion within the glass ceramic, which has an almost crystalline structure. The newly formed crystalline phase is considered metastable owing to its broad diffraction peak width, which poses challenges for applying Rietveld refinement without a hypothesis. To address this, we conducted XRD calculations for structurally stable configurations verified via self-consistent field calculations using density functional theory (see Fig. S1). Several initial structures were assumed, and insertion and substitution-type structural models were obtained based on the LGPS structure, as depicted in Fig. 4. As shown in the figure, the insertion type involves the presence of Br^−^ ions within the gaps between PS_4_ molecules. Given that the PS_4_ molecule carries a charge of − 3 and the GeS_4_ molecule in LGPS structure is − 4, there are likely three or four Br^−^ ions associated with each PS_4_ molecule. Br^−^ ions tend to form clusters together with Li ions.

**Figure 5:**
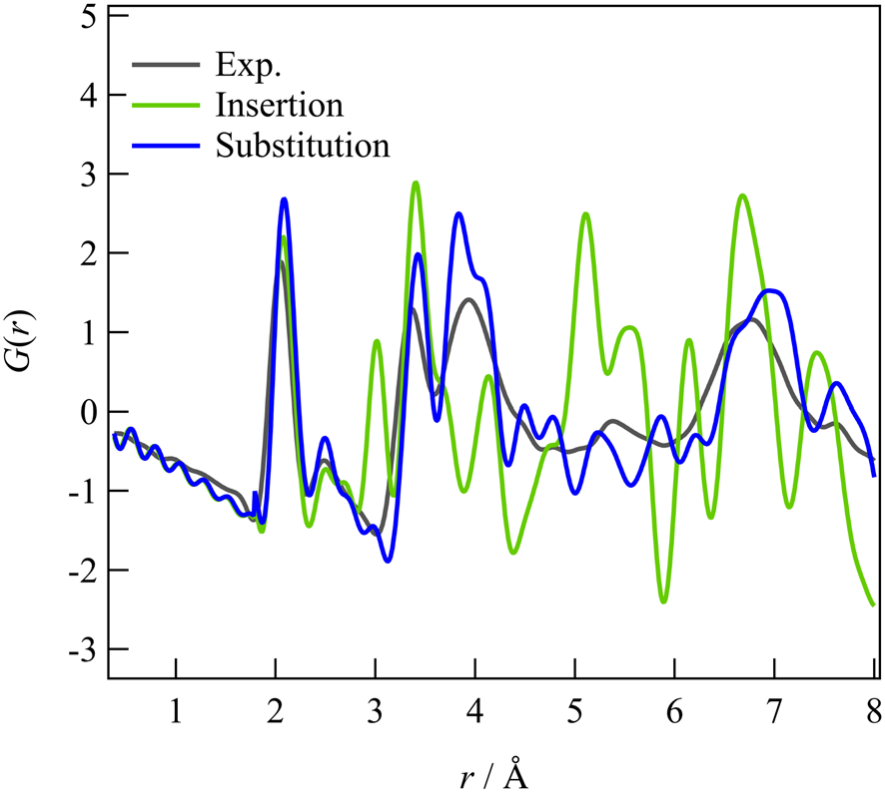
Comparison of *G*(*r*) calculations and experimental data for insertion type (**a**) and substitution-type (**b**) structural models of Br-doped glass ceramic.

Figure S1a represents the structure without Br, while Fig. S1b shows the addition of one Br atom to both the (0,0,0) and (0.5,0.5,0.5) sites. The circle in the in the figure represents the experimental XRD, while the blue line represents the calculated XRD based on the structural model. One Br added to the model results in an approximate LiBr ratio (mol%) of 7.7. Considering a more realistic scenario, we added approximately two Br ions, resulting in a LiBr ratio of ~ 14.3 mol%. Additionally, we investigated the substitution-type structural model using the same approach. Four S ions at positions (0.00000, 0.30099, 0.79168), (0.00000, 0.69901, 0.79168), (0.20443, 0.50000, 0.59727), and (0.79557, 0.50000, 0.59727) on the P1 site were replaced with one to four Br^−^ ions, as shown in Fig. S1c–e. As depicted in Fig. S1f,g, we examined structural models for insertion and substitution types. The results indicated that the substitution-type with four Br atoms at the P1 site closely matched the experimental data. The PDF *G*(*r*) for these two models was calculated and compared to validate the insertion and substitution types in terms of local structure. The experimental data are represented by black lines, the calculated insertion type by light green solid lines, and the substitution-type by blue solid lines (see Fig. 5). It is evident from these figures that the peak reproducibility is higher for the substitution-type compared to the insertion type. Furthermore, the substitution-type structural model accurately reproduced the characteristic peak appeared at ~ 4.0 Å, confirming its presence in the system with Br. Therefore, it is highly likely that Br forms clusters at the P1 site.

**Figure 6:**
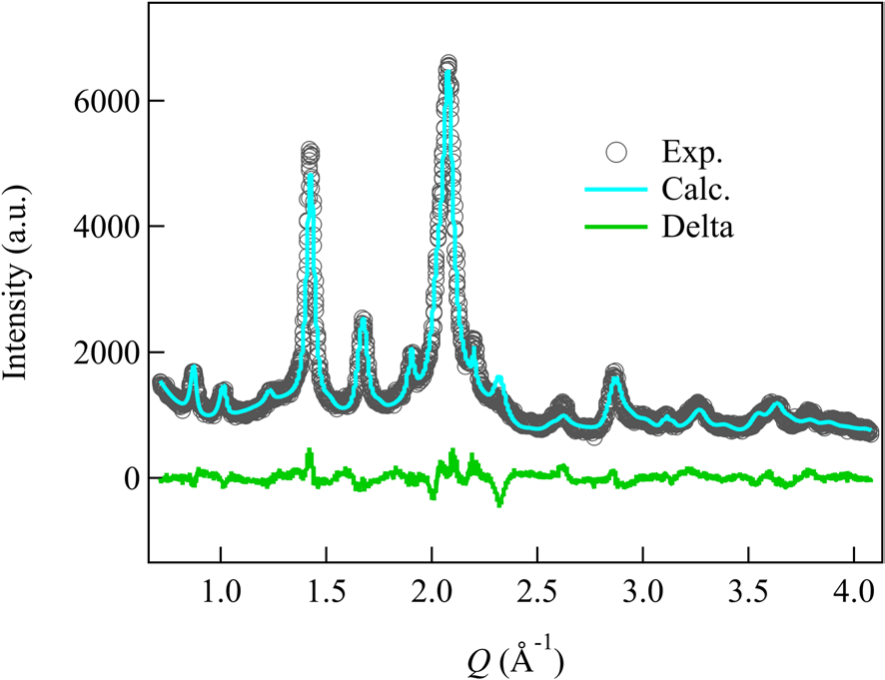
Rietveld refinement results using a substituted structural model.

Based on our consideration, we chose the substitution-type structural model and replaced the P1 site with a Br cluster. Structural optimization was then carried out using Rietveld analysis. The results revealed that the occupancy of Br is 0.15 ~ 0.25, suggesting that 15 ~ 25% of PS_4_ molecules are replaced by the Br cluster. Figure 6 shows that incorporating an 8% Br cluster in the structural model accurately reproduces the XRD pattern. Detailed structural parameters can be found in Table S1. Notably, the atomic displacement of P in the Br cluster differs significantly from the unsubstituted structural model, indicating substantial anion fluctuations within the structure compared to the pure unsubstituted model.

**Figure 7:**
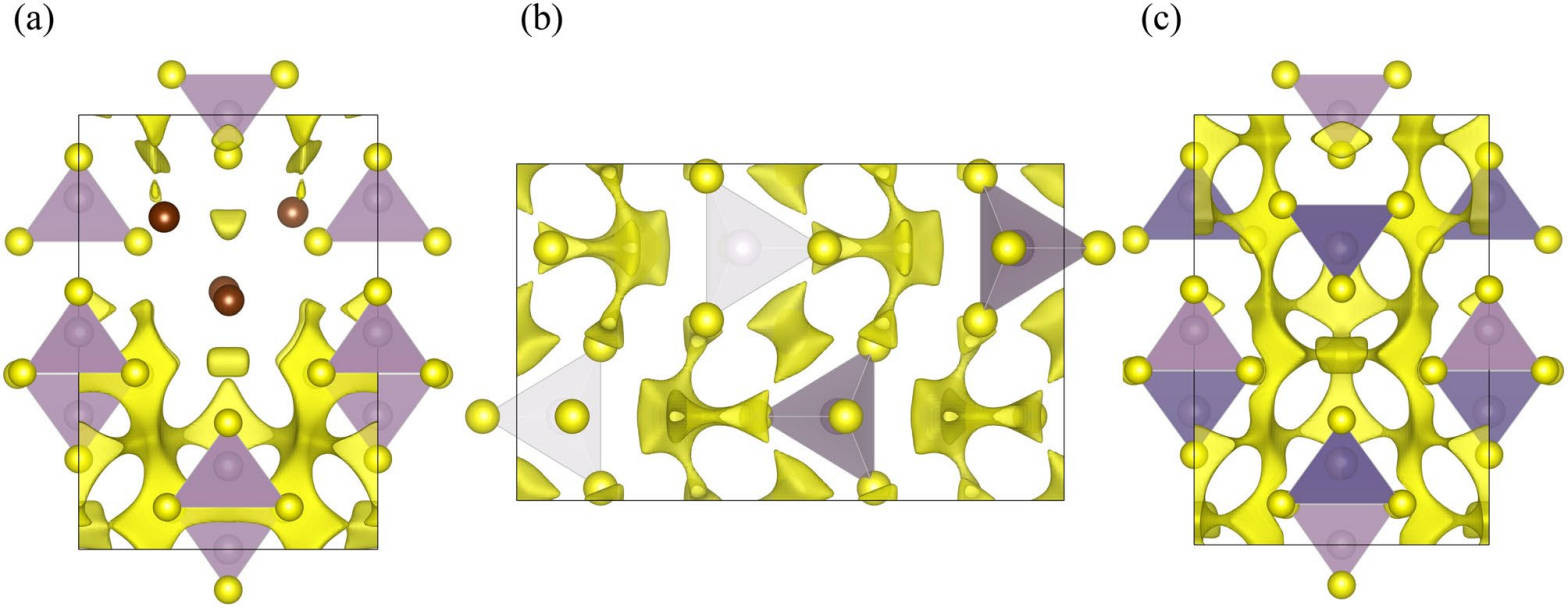
Visualization of Li-ion conduction paths using the Bond Valence Sum method. (**a**) Br-substituted LiPS in this work, (**b**) β-Li_3_PS_4_, and (**c**) LGPS systems.

Figure 7a illustrates the Li-ion conduction pathway investigated by depicting the Δ*E* = 0.80 eV in the Br cluster-substituted structural model. The presence of Br clusters hinders the pathway for Li-ion conduction, while the pathway emerges in regions without Br. Upon examining the overall structural model, it is evident that the paths do not form in the *c*-axis direction but rather in the *b*-axis direction. The calculations for β-Li_3_PS_4_, which exhibits low conductivity, yield similar results as depicted in Fig. 7b, indicating the absence of such pathways. In contrast, the structural model of LGPS with high ionic conductivity displays a formation of a three-dimensional pathway, as depicted in Fig. 7c. The presence of Br is considered a contributing factor to the enhanced ionic conductivity through halogen addition. However, the limited formation of high-conductivity pathways along the *c*-axis in the vicinity of Br likely accounts for the disparity in ionic conductivity compared to LGPS.

## Conclusion

Br-doped Li_3_PS_4_ exhibits a minimal presence of P_2_S_7_ molecules that effectively suppresses the generation of H_2_S. Instead, it forms an LGPS-like structure and demonstrates high ion conductivity. This characteristic makes it a promising material for solid electrolytes in all-solid-state batteries, striking a balance between desirable properties.

The local structural characterization conducted through high-energy XRD and PDF analysis confirms that the molecular framework structure of the precursor glass remains unaltered. Upon converting to glass ceramic and subsequent crystallization, PS_4_ sites randomly incorporate Br atoms. Furthermore, using the Bond valence sum method to visualize Li-ion conduction pathways in the structural model, one can observe that Br facilitates the formation of ionic conduction paths resembling those found in LGPS. However, the formation of pathways along the *c*-axis direction is insufficient owing to structural geometry limitations.

This study emphasizes the importance of structure control in glass ceramic as it can pave the way for developing novel glass/glass–ceramic solid electrolyte materials in future research.

### Supplementary Information

Supplementary Information.

## Data Availability

The datasets generated and/or analyzed during the current study are available from the corresponding author on reasonable request.
