# Supplementary material for: Structural analysis and ionic conduction mechanism of sulfide-based solid electrolytes doped with Br
Source: Sci Rep. 2023 Sep 25;13:16063. doi: 10.1038/s41598-023-43347-9 (PMC10520000; doi:10.1038/s41598-023-43347-9)
Supplement: Supplementary file 1 — Supplementary Information. [file 41598_2023_43347_MOESM1_ESM.docx]

**Supplementary information**

**Structural Analysis and Ionic Conduction Mechanism of Sulfide-Based Solid Electrolytes Doped with Br**

^1,2^Hiroshi Yamaguchi, ^3^Kentaro Kobayashi, ^3^Satoshi Hiroi, ^2^Futoshi Utsuno, and ^1,3,4^Koji Ohara*

*^1^Graduate School of Natural Science and Technology,1060, Nishikawatsu-cho, Matsue, Shimane 690-8504, Japan*

*^2^Idemitsu Kosan Co. Ltd., 1280, Kamiizumi, Sodegaura-city, Chiba 299-0293, Japan*

*^3^Faculty of Materials for Energy, Shimane University, 1060, Nishikawatsu-cho, Matsue, Shimane 690-8504, Japan*

*^4^Diffraction and Scattering Division, Japan Synchrotron Radiation Research Institute, 1-1-1, Kouto, Sayo-cho, Sayo-gun, Hyogo 679-5198, Japan*

The supplementary information file includes the below contents:


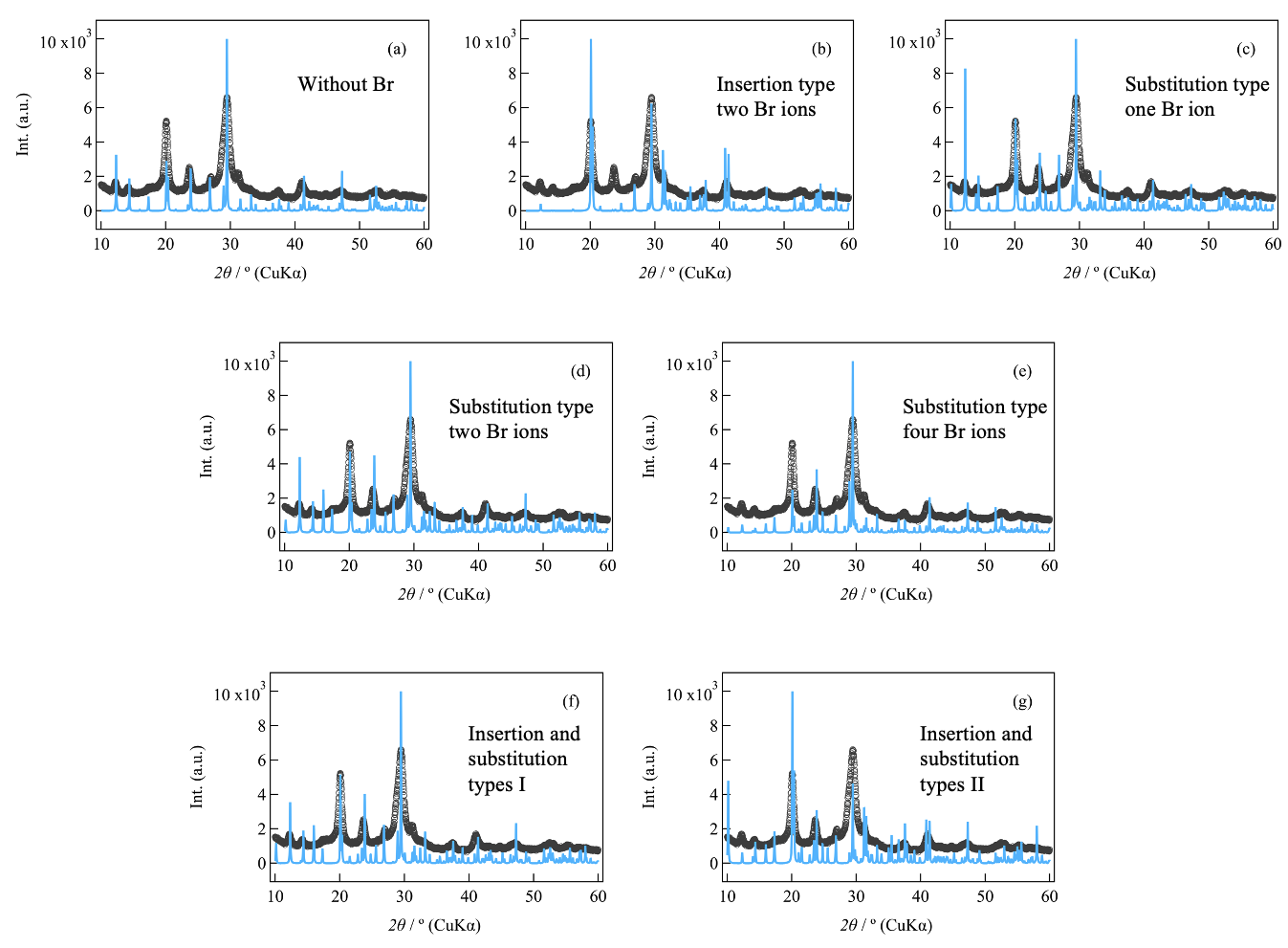


Figure S1: Comparison of the XRD patterns of (a) without Br, (b) insertion type, (c, d, e) substitution-type, and (f, g) insertion-substitution type structure models of glass ceramic (circle: experimental data, blue line: calculated data)

Table S1：Detailed Structural parameters of model incorporating 8% Br cluster

| Atom | x/a | y/b | z/c | Occ. | B |
| --- | --- | --- | --- | --- | --- |
| P | 0.000 | 0.500 | 0.690 | 0.920 | 2.000 |
| P | 0.000 | 0.000 | 0.500 | 1.000 | 2.000 |
| S | 0.000 | 0.200 | 0.428 | 0.853 | 5.000 |
| S | 0.000 | 0.252 | 0.085 | 0.754 | 5.000 |
| S | 0.000 | 0.682 | 0.781 | 0.789 | 5.000 |
| Br | 0.000 | 0.200 | 0.428 | 0.147 | 5.000 |
| Br | 0.000 | 0.252 | 0.085 | 0.246 | 5.000 |
| Br | 0.000 | 0.682 | 0.781 | 0.211 | 5.000 |
| Li | 0.026 | 0.270 | 0.187 | 0.474 | 0.077 |
| Li | 0.000 | 0.500 | 0.947 | 0.890 | 0.039 |
| Li | 0.747 | 0.247 | 0.000 | 0.720 | 0.149 |
| Li | 0.000 | 0.000 | 0.251 | 0.770 | 0.076 |
| *P*4 2/*nmc*, *a* = 8.7541, *c* = 12.521, *R*_wp_ = 6.68%, *R*_e_ = 2.73%, χ^2^ = 5.9404, impurity: 8.1 wt% (Li_2_S) | | | | | |
